# Effects of comorbid disorders on reward processing and connectivity in adults with ADHD

**DOI:** 10.1038/s41398-021-01758-0

**Published:** 2021-12-16

**Authors:** Oliver Grimm, Daan van Rooij, Asya Tshagharyan, Dilek Yildiz, Jan Leonards, Ahmed Elgohary, Jan Buitelaar, Andreas Reif

**Affiliations:** 1 Department of Psychiatry, Psychosomatic Medicine and Psychotherapy, University Hospital, Goethe University, Frankfurt, Germany; 2 Donders Centre for Cognitive Neuroimaging, CNS Department, University Medical Centre Nijmegen, Nijmegen, Netherlands

**Keywords:** Human behaviour, Diagnostic markers, ADHD, Neuroscience

## Abstract

ADHD is a neurodevelopmental disorder with a long trajectory into adulthood where it is often comorbid with depression, substance use disorder (SUD) or obesity. Previous studies described a dysregulated dopaminergic system, reflected by abnormal reward processing, both in ADHD as well as in depression, SUD or obesity. No study so far however tested systematically whether pathologies in the brain’s reward system explain the frequent comorbidity in adult ADHD. To test this, we acquired MRI scans from 137 participants probing the reward system by a monetary incentive delay task (MIDT) as well as assessing resting-state connectivity with ventral striatum as a seed mask. No differences were found between comorbid disorders, but a significant linear effect pointed toward less left intrastriatal connectivity in patients depending on the number of comorbidities. This points towards a neurobiologically impaired reward- and decision-making ability in patients with more comorbid disorders. This suggests that less intrastriatal connectivity parallels disorder severity but not disorder specificity, while MIDT abnormalities seem mainly to be driven by ADHD.

## Introduction

ADHD is a neurodevelopmental disorder, primarily characterized by inattentiveness, impulsiveness and hyperactivity [1]. Prevalence in children is between 5 and 7% world wide, with a high persistence into adulthood [1]. A high number of childhood patients report persistent symptoms and impairment in adulthood, leading to prevalence ranges between 2.5 and 3% in adults [2–4]. Apart from impairment due to ADHD symptoms, patients also have an increased chance of developing comorbid psychiatric disorders over the lifespan. Affective disorders, personality disorders, and substance use disorders (SUD) are all significantly more prevalent in patients with ADHD as compared to the general population [5–7]. Patients suffering from ADHD and comorbid disorders report higher disease burden [5] and decreased treatment efficacy [8], underlining the need for more research into the underlying mechanisms resulting in this high comorbidity for subjects with ADHD.

One of the functional brain networks that potentially links the emergence of ADHD and comorbid disorders is the reward system. Extensive literature demonstrates subjects with ADHD show altered behavior and neural activation patterns during both the anticipation and reward of receipts [9, 10], in frontal-striatal brain regions classically associated with reward processing. In particular, the nucleus accumbens (NAcc) is a central region for the processing of rewards in humans as well as in rodents [11, 12], and is a key node in the alterations of the reward system observed in ADHD [13–15].

An important feature of the comorbid disorders mentioned above is that each of them is also associated with altered reward processing, as recently discussed [10]. Hence the central hypothesis of the present study is that neural alterations of reward system may serve as the biomechanical feature explaining the link between ADHD and these comorbid disorders.

Therefore, In the current study, we investigate the reward network in subjects suffering from ADHD and various comorbid disorders where alterations in the reward network have been suggested to play a role, namely major depressive disorder (MDD), substance use disorder (SUD), and obesity. Previous neuroimaging research in MDD, obesity, and SUD point to the reward system as common denominator [10]. For SUD, the reward system is an obvious main target, as almost all drugs directly impact on the reward system. MDD with its loss of motivation, drive, and pleasure, has been linked to a blunted reward anticipation in the MID task [16], as well as other dopaminergic deficits [17]. Obesity has been recently conceptualized as a variant of a SUD, because primary enforcers like food elicit a strong dopaminergic response [18]. A blunted reactivity to food cues in the caudate predicted weight gain over 6 months [19].

We will investigate both functional brain activation and functional connectivity. For the former, we will use the Monetary Incentive Delay task [20, 21] to measure neural activation during the anticipation and receipt of monetary rewards in subjects with ADHD and these disorders. For the latter, resting-state fMRI measures of all these subjects will be acquired. Resting-state fMRI will be used to calculate functional connectivity from the nucleus accumbens as a seed region-of-interest (ROI) to other parts of the brain.

We address three research questions. (1) Is there a general effect of comorbidities on MID task performance and neural activation and connectivity? (2) Is there a dose effect of comorbidities? (3) Is there a specificity effect of comorbidities? We hypothesize that subjects with ADHD and comorbid disorders show a larger impairment on MID performance and altered neural activation patterns than patients suffering from ADHD alone, in particular in the NAcc. We also expect altered functional connectivity with the NAcc region in subjects with comorbidities. We further expect that multiple comorbidities will be associated with larger impairments. We hypothesize that subjects with comorbid MDD will show decreased reward-related speeding/sensitivity to reward, and hypoactivation during reward anticipation, while for subjects with SUD and obesity we expect stronger reward sensitivity and hypoactivation during reward receipt.

## Methods

### Participants

The study includes *n* = 137 physically healthy subjects (for demographics see Table 1). Recruitment took place at the Donders Centre for Cognitive Neuroimaging, Nijmegen, Netherlands, and the Goethe University Frankfurt am Main.

**Table 1:** Demographic overview with behavioral measures of task.

	MID task sample		Resting-state sample	
	ADHD	ADHD with comorbid disorders	ADHD	ADHD with comorbid disorders
Number of participants	32	100	29	108
Age	27.16 (6.64)	33.38 (9.10)	25.97 (5.91)	33.56 (8.96)
Site				
Nijmegen	13	44	10	43
Frankfurt am Main	19	56	19	65
Sex				
Female	16	56	15	60
Male	16	44	14	48
Number of ADHD symptoms (mean/stdev)	7.71 (6.76)	5.69 (5.56)	8.9 (6.19)	6.56 (6.78)
Number of comorbidities				
1		54		55
2		34		41
3		12		12
Type of comorbidities				
Overweight		61		66
Depression		59		64
Substance addiction		39		43
Subgroups				
Overweight		22		25
Depression		18		17
Substance addiction		14		13
Overweight and depression		21		23
Overweight and substance addiction		4		6
Depression and substance addiction		9		12
Overweight and depression and substance addiction		12		12
Medications				
Stimulants		34		35
Atomoxetine		2		2
Antidepressants		10		11
Other		1		1
Mean response time (ms)				
Winning condition	207.67 (28.82)	211.17 (33.66)		
Control condition	220.48 (32.55)	220.98 (41.28)		
Mean number of omission errors				
Winning condition	0.41 (0.55)	0.6 (0.98)		
Control condition	1.56 (4.15)	1.23 (3.67)		

The demographics and clinical characteristics are given for the MID sample and the connectivity sample. Standard deviations are given in brackets.

Inclusion criteria were (1) age between 18 and 50 years; (2) sufficient understanding of the Dutch/German language, (3) established childhood diagnosis of ADHD made by a specialist (DSM-IV criteria) and additional validated ADHD-questionnaires (self-report, CAARS, Wender-Utah scale >30 points), and (4) additional comorbid condition of depression (according to DSM-IV), alcohol- or amphetamine-dependence (DSM-IV), or overweight (BMI > 25 kg/m^2^). As we had mainly access to patients who used alcohol and amphetamine as primary drug of abuse, included these as major inclusion criteria. Other patients who were screened were excluded because of polytoxicomania.

Exclusion criteria were other mental illnesses (apart from ADHD, depression, and SUD), serious acute or chronic physical diseases, pregnancy, as well as exclusion criteria of the MRI examination. Only patients with at least 4 weeks of stable medication regimen were included. Stimulants, alcohol, and nicotine were stopped on the day of the scan. Patients with antipsychotic medication were excluded. Participants were examined by a registered psychiatrist in Frankfurt in a specialized ADHD-outpatient clinic. In Nijmegen selection and diagnostic procedures were conducted by trained psychiatrists or psychologists. No inpatients participated in the study. No inpatients did participate in the study.

For assessment of psychosocial functioning, a blinded and trained psychiatrist rated in the Frankfurt dataset the global assessment of functioning (GAF) and the clinical global impression (CGI) score by clinical interview.

The project was carried out in accordance with the provisions of the Declaration of Helsinki (World Medical Association 2013) and the European guidelines on Good Clinical Practice and was approved by the Ethics Committee of the Medical Faculty of the J.W. Goethe University Frankfurt am Main (reg.no. 256/16) and in Nijmegen by the Radboud University (reg.no. ABR64162). The study was registered in the German register of clinical studies on 16/11/2016 with the study ID DRKS00011248, indexed in the WHO clinical trial search platform (https://apps.who.int/trialsearch/). Subjects gave written informed consent. The subjects received 10€ per hour for participation. In addition, the monetary gain of the Monetary Incentive Delay Task of all three measurement dates was paid out to the volunteers.

### Monetary incentive delay fMRI-paradigm

**Fig. 1:**
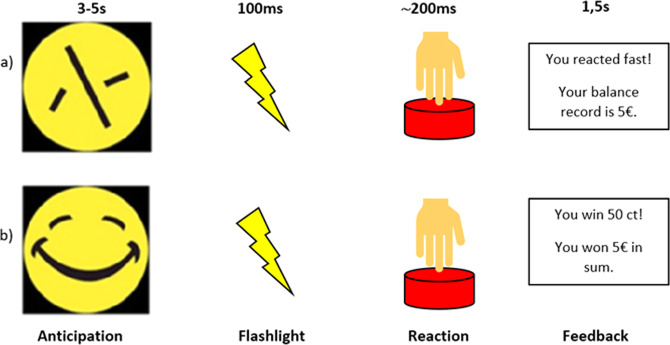
Monetary incentive delay (MID) task. Event-related functional magnetic resonance imaging (MRI): 30 control conditions (**a**) and 30 winning conditions (**b**). In sum 60 trials. A circle (**a**) or a smiley (**b**) is displayed in the anticipation phase. Then a flashlight appears and the participant has to react as fast as possible by pressing a button. After the reaction, a feedback is shown: control condition (**a**): feedback about the success of the reaction and winning condition (**b**): feedback about winning money—50 cents or a booster of 2€.

We used a MID-task in combination with fMRI measurement. A modified version of this MID-task has been evaluated extensively before and leads to a valid and reliable signal in the striatum during anticipation. The task was validated in previous studies [21, 22]. Volunteers were shown 30 “smileys” as well as 30 neutral, scrambled “control smileys” on a screen in the MRI in an unpredictable order, to which they had to react as quickly as possible with the push of a button after a flashlight occurred. After presentation of the smileys (or conditioned stimuli), the participants earned monetary feedback of 50 cents if they reacted quickly. In order to prevent habituation, participants were unexpectedly rewarded with a booster prize (max. four times) of 2€ in between. If the reaction time was too slow, the participant did not win money. The smiley represents the winning condition (b). In a control condition (a) the test persons were shown a scrambled smiley, a yellow circle (see Fig. 1). In the control condition only a written positive or negative feedback was provided. The money won, as well as the current account balance, was displayed on the screen after each run (e.g., “You win 50 cents, the account balance is 10 euros”).

After each trial, the reaction time was adapted depending on success (tougher next trial, reaction time +10%) or miss (easier next trial, reaction time +5%). In order to increase the expectation of rewards, the participants were informed beforehand that money won in the MID task will be paid out in cash directly after the measurement.

The behavioral measures (reaction times win, control, win minus control, omission error win, omission errors control, sum of money won) were tested for the same models as the fMRI analysis in an ANOVA model in SPSS 25. First, we corrected for age, sex, and site and compared with and without comorbidity. Second, we defined the number of comorbid disorders as ordinal variable and included it as a factor (while adjusting for age, sex, and site), and third, we defined a factor for all combinations of different comorbid disorders.

### Data analysis: fMRI preprocessing

Images from participants were realigned, slice-time corrected, spatially normalized to standard stereotactic space (Montreal Neurological Institute [MNI] template), resampled to 3 mm isotropic voxels, and smoothed with 8 mm full-width at half-maximum Gaussian kernel. A band-pass filtering was used in the frequency band frequency bands to 0.01–0.1 Hz to get rid of non-neural signals for the resting-state data. Further noise correction was done by regressing out motion parameters derived from the realignment procedure, the 1st order derivative of movement parameters. Data analysis was done with SPM12 in addition to CONN toolbox for preprocessing of resting-state data. A band-pass filter reduced frequency bands to 0.01–0.1 Hz, additional noise correction of rs-fMRI was done by regressing out motion parameters (from rigid-body transformation), their 1st order derivative and correcting for cerebrospinal-fluid signal and white-matter-signal (so-called aCompCor-strategy) [23].

### Accumbens seed connectivity

**Fig. 2:**
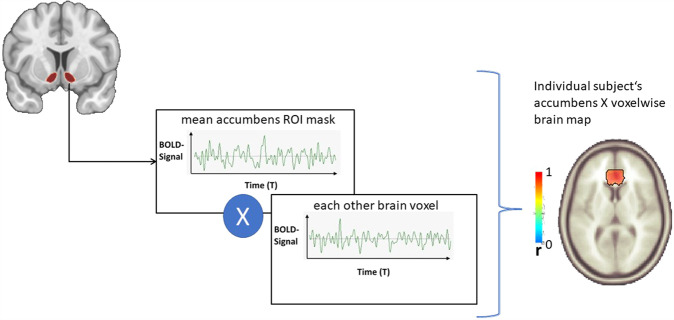
Resting-state functional connectivity. Generating BOLD-response time series for specific brain regions (ROIs). The time series were correlated with BOLD-responses from the rest of the brain. In this example, the BOLD-response in the selected ROI (nucleus accumbens) shows a positive correlation with the BOLD-response in the anterior division of the cingulate gyrus. A significant overlap in BOLD-responses indicates functional connectivity between the compared brain regions as measured by a z-normalized correlation coefficient.

For seed-voxel connectivity (see Fig. 2), we used the ROI mask of the nucleus accumbens from the high-resolution probabilistic in vivo atlas of human subcortical brain nuclei (CIT168) [24]. Data processing was done with CONN v1.8 toolbox with SPM12.

### Data analysis: group statistics

Sample size was calculated for a ANOVA analysis with four groups and at least one covariate for site. We estimated effect sizes from a meta-analysis of the MID task 7 (*d* = 0.58). For an alpha error of 0.05 and a power of 0.8, a sample size of *n* = 136 was calculated. For the simpler comparison ADHD with versus ADHD without comorbid disorders, a sample size of *n* = 76 was calculated. For the regression model with the number of comorbid disorders, a sample size of *n* = 81 was calculated. Sample size calculations were done with GPower Version 3.1 [25].

First-level correlation maps were calculated by extracting the residual BOLD-time course from the seed ROIs and correlating these with the other voxels within the brain. These correlation coefficient maps were then converted via a normally distributed z-score (Fisher transformation). Transformed correlation maps were used for directed paired *t*-tests on the second-level stage. For correction of multiple testing during second-level statistics, we used cluster-wise whole-brain analysis with topological FDR correction pFDR<0.05 (cluster defining voxel threshold *p* < 0.001) [26].

We defined three hypotheses to test for the effects of comorbidity. First, we compared patients with and without comorbidity. Second, we looked at a linear effect of comorbidity (no, one, two, or three, equivalent to a T-test vector of [−3 −1 1 3]). Third, we compared via F-test contrast all 8 subgroups of different combinations of depression, SUD, or obesity. All tests included age, gender, and site as covariates. The first two tests were defined as *t*-tests, the third one as F-test.

We calculated differences in medication between the comorbidity subgroups with an univariate general linear model in SPSS. The comorbidity (SUD, MDD, or overweight) was used as dependent variable, whereas medication type (stimulant, atomoxetine, antidepressant, none, other) was used as independent variable with age, sex, and site as covariates.

## Results

### Clinical symptom

A two-sample *t*-test revealed a trend-wise non-significant difference for the number of ADHD symptoms in only ADHD vs. ADHD with comorbidity with a *p*-value of *p* = 0.09 (t(130) = 1.69) for the MID-sample, and *p* = 0.09 (t(135) = 1.67) for the rs-fMRI-sample.

Depressive disorders were significantly associated with more antidepressant use (*p* < 0.001, F(1,129) = 14.69), stimulants and other medication did not differ among groups.

### Monetary incentive delay task

Neither the direct comparison between “pure” ADHD and ADHD with comorbidity, the test of linear effects on the number of comorbidities nor the comparison among groups yielded any significant result for the analysis of the activation in the ROI for the main contrast anticipation WIN > anticipation CONTROL. The behavioral data did not reveal any significant differences for the hypotheses mentioned above either (*p* > 0.06, F < 3.56).

We extracted the mean beta from the accumbens-ROI-mask and correlated it to GAF and CGI in *n* = 51 patients (details are given in supplemental Table 1). GAF was not significantly correlated (*p* = 0.63, *r* = 0.069), but CGI (*p* = 0.035, *r* = −0.29) was significantly correlated with the extracted task-betas.

To test whether a certain ADHD dimension specifically influenced our results, we extracted the number of symptoms and correlated these with the number of symptoms with extracted mean beta from the nucleus accumbens for the MID task. Neither inattention (*p* = 0.9, *r* = −0.013) nor hyperactivity (*p* = 0.69, *r* = 0.04) were significantly correlated with the mean extracted beta.

### Accumbens seed connectivity

The difference between ADHD without and with comorbid disorder did reach FDR-corrected significance when using a lower *p*-threshold (*p* < 0.005, MNI +2/+56/+34, *k* = 534, pFDR = 0.001). However, we did not use *p* = 0.005 for cluster-threshold definition because of recent critique and controversy [27].

The comparison of accumbens seed connectivity in dependence of the number of comorbidities showed a significant decrease in FC between accumbens and parts of the left putamen and the left insula (pFDR=0.002, *k* = 433, MNI −30/−6/+14, T(130) = 3.37).

There was no effect when comparing among all ADHD and comorbidity subgroups.

We extracted the mean beta from the significant FC-cluster and correlated it to GAF and CGI in *n* = 54 patients (details are given in supplemental table 2). Neither GAF (*p* = 0.55, *r* = 0.82) nor CGI (*p* = 0.068, *r* = −0.25) were significantly correlated with the extracted task-betas.

In order to test whether a particular ADHD dimension specifically influenced our results, we extracted the number of symptoms. We correlated the number of symptoms with the extracted mean beta value of the significant cluster in the left putamen in the rs-fMRI analysis. None of inattention (*p* = 0.97, *r* = −0.004) or hyperactivity (*p* = 0.91, *r* = 0.01) were significantly correlated with the mean extracted beta value.

## Discussion

**Fig. 3:**
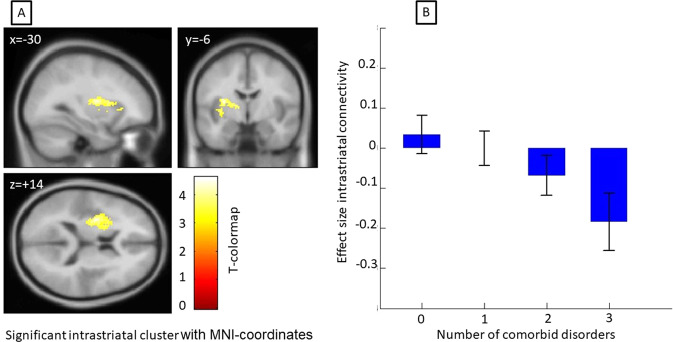
Change of accumbens seed connectivity in dependence of number of comorbidities. **A** The intrastriatal cluster in the left striatum which significantly demonstrated less connectivity to the accumbens dependent on the number of comorbid disorders. The color coding of the cluster is given in the T-colormap. **B** The decline of connectivity from the intrastriatal cluster to the accumbens in for each number of comorbid disorders. Effect sizes were extracted from the significant cluster from panel B.

Our results failed to find a differential effect of reward anticipation in the MID task for patients with and without comorbidity, neither on a behavioral nor neural response level. Neither number of comorbid disorders nor different subgroups were delineated by neural activation in the MID task. This is in contrast to our primary hypotheses and to findings from previous studies looking at only one of the comorbid disorders [10]. While NAcc-connectivity did only change trend-wise between the with/without group, the intrinsic striatal connectivity was lower or blunted depending on the number of comorbidities (see Fig. 3A, B). Interestingly, specific comorbid disorders were not significantly different, while only the linear number of comorbidities was. This argues for a disorder-unspecific effect of comorbidities on reward network activation, and for a more general effect of load of psychopathology.

In general, existing functional imaging research on subjects with ADHD and comorbid MDD are scarce, but those that have been performed indicate that the depressive symptoms were driving neural activation during a MID task, leading to lower ventral striatum activation during reward anticipation in ADHD patients with more depressive symptoms [28]. Literature on the MID task in ADHD and comorbid SUD show mixed results in reward anticipation, based on different substances and treatment states [29]. Regarding the literature on ADHD and obesity, one study observed that the link between the ADHD polygenic risk score and both impulsivity and BMI was associated with striatal activation during the MID task [30]. However, no reward-related imaging studies including both subjects with ADHD and obesity have been performed so far. Coming from this background, a decrease in intrinsic striatal connectivity might be interpreted as blunting of the reward system. However, this is contradicted by the failure to detect a linear effect of comorbidity on reward anticipation in the MID task. There are some potential interpretations: First, the MID task is designed to get a strong main task effect as documented by, e.g., high intra-session reliability. Such a high task effect might lead to a strong ceiling effect precluding the detection of group differences. The NAcc-connectivity might be more sensitive but is more difficult to interpret as previous studies reported both increased as well as decreased connectivity patterns. The connectivity between NAcc and the PFC is often discussed in terms of a cognitive top-down control of emotions and motivational drives. This has been found to be related to ADHD and delay-discounting impulsivity [14], but was not significant in our study. Therefore, our study does not support PFC-NAcc-connectivity as a common pathomechanism in ADHD and comorbid disorders. A recent study [31] did not detect differences between ADHD and controls but a correlation between increased striatal connectivity and impulsivity scores. While this study underscores the advantages of linear measures, it is not entirely comparable to our protocol, as the comorbidities in our study are not necessarily related to impulsivity, but to a general increase of disorder-related impairment which can be found even in low impulsivity patients.

Our study finds a disturbance of the striatal connectivity but no abnormalities in the task-based reward anticipation. Decision and reward learning, however, have other aspects than reward anticipation, only. In a study on 349 adolescents, striatal connectivity was shown as the neural equivalent of a general decision-making component, the “decision acuity”, which also seems to show a general connection with psychopathology [32]. It is a plausible assumption that this is directly related to ADHD and the comorbid disorder’s underlying dysregulation. Future studies should in addition to striatal connectivity look at behavioral phenotypes related to, e.g., decision acuity.

To better understand clinical significance of our findings, we correlated the betas from the significant FC cluster with proxies for psychosocial functioning, namely GAF and CGI in a subset of the sample. This was only significant for the MID-task (and trend-wise for the connectivity results) when not accounting for the number of two tests, so it suggests that a lower activation and connectivity goes together with a lower CGI. In keeping with this observation, CGI but not GAF was correlated with the number of comorbid disorders. We were not able to find a correlation between a specific symptom dimension of the DSM-scale in our sample. While this points toward an effect non-specific for ADHD, a limitation is the fact that in an all ADHD sample the range of symptoms is necessarily low as per definition five or symptoms have to be met for diagnosis. The fact that we find a difference in CGI but not ADHD symptom scores between comorbid groups emphasizes the need to take comorbid disorders into consideration when studying ADHD, as their presence may influence the severity of a disorder even when the base number of symptoms remains similar.

A general limitation of our study is its focus on categorical categories. Other studies like the ABCD-study or the IMAGEN-cohort looked longitudinally at more fine-grained psychopathological dimensions. However, the absolute number of cases with full-blown comorbid diagnoses are low and SUD diagnosis is rare in children and adolescents. Studies which draw samples from the general population need a much higher sample size to sample enough participants in order to have participants with a high load of psychopathology. In contrast to this, our study may not be representative, but was able to recruit ADHD patients with a complex comorbidity pattern. Another strength of the current study is that we can combine multiple imaging measures in the same well-defined sample. This allowed us to look at the neural response of the reward network both from a task-based and resting-state perspective. Our results indicate that subtle alterations of the reward network may be visible in resting state but not picked up by reward-related functional imaging tasks, which is an important caveat to take into consideration when researching reward network functioning in patient groups.

More specific limitations are the lack of a healthy control group. While our paradigms have been established in ADHD [9, 15, 33] and validated for time-dependent reliability [34], we did not feel that a healthy control group would have added more scientific value, as our main objective was not to test differences between healthy participants and ADHD patients. However, it might have served as a baseline for effect sizes. In addition, we did not include groups without ADHD and the comorbid disorders. This would have enabled us to test for any ADHD by comorbid disorder-specific interactions and ADHD-independent effects. Future studies might use such a comparison, e.g., ADHD, ADHD + depression, and depression-only.

However, the most interesting conclusion comes from the lack of specific diagnosis-related diagnosis-related groups. This is countered by—at least in the accumbens-connectivity analysis—a linear decrease of intrastriatal connectivity with the numbers of comorbidity. This points to some interesting transdiagnostic effects: Is number of comorbidity related to a general drop in psychosocial functioning and general severity of illness? In our study, we did not have access to individual data which would allow to answer this question empirically. So far, studies with more fine-grained dimensional measures in contrast to categorial disorders did not demonstrate decisive advantages or differences when trying to understand the underlying neurobiology: Some of these issues were discussed in more detail in a recent review [10]. Since it was not specific psychopathology but rather the number of syndromes that revealed lower intrastriatal connectivity, the question arises whether we are dealing with a general psychopathology factor. The meta-analytic evidence for transdiagnostic neuronal abnormalities in clinical samples suggests in principle that there is a correspondence between psychiatric disorders and their neuronal dysregulation and neuronal abnormalities associated with general psychopathology. In particular, many of the observed disorders appear to converge at key nodes of the salience network, with the degree of dysfunction correlating with psychopathology. Intrastriatal connectivity—as found in our study—can be interpreted as a subset of the salience network. In a study looking at developmentally derived growth charts of striatal connectivity, ADHD symptoms as well as general psychopathology were linked to an age-delayed connectivity between the ventral striatum and different parts of the striatum [35]. While this fits well with our finding of decreased intrastriatal connectivity, the comparability of this much younger sample (maximum 22 years) to our older sample is difficult. A recent paper which demonstrated a decreased connectivity within the salience network in schizophrenia and psychotic bipolar disorder demonstrates that the neuronal phenotype of a decrease in striatal connectivity is not disorder-specific but points to a dysregulation linked to general psychopathology [36]. The use and analysis of such a general psychopathology component may be important not only in the search for transdiagnostic markers of psychiatric risk, but also in the study of pathophysiological mechanisms [37].

In conclusion, we demonstrate functional connectivity alterations with the NAcc in subjects with ADHD and comorbid MDD, SUD, or obesity, as a function of the number of comorbidities. No specific effects of any one comorbidity were detected, and neither performance nor neural activation during the MID task was influenced by comorbidities. Our study is more in line with a general dysregulation of the reward network, linked to disease severity which is independent of a specific disorder. This was found for accumbens-connectivity but not for the reward anticipation task. While this task has been able to detect blunted reward anticipation before [9], other studies found opposite effects of ADHD and depression or alcohol abuse.

## Supplementary information

supplementary table
