# Supplementary material for: Effects of comorbid disorders on reward processing and connectivity in adults with ADHD
Source: Transl Psychiatry. 2021 Dec 16;11:636. doi: 10.1038/s41398-021-01758-0 (PMC8674233; doi:10.1038/s41398-021-01758-0)
Supplement: Supplementary file 1 — supplementary table [file 41398_2021_1758_MOESM1_ESM.docx]

**Supplemental table 1**

|  | | comorbid disorder | | | | | |
| --- | --- | --- | --- | --- | --- | --- | --- |
|  |  | Only ADHD | | | ADHD with comorbid disorder | | |
|  |  | Mean | standard deviation | number | Mean | standard deviation | number |
| Age | | 27.15 | 6.21 |  | 32.26 | 8.68 |  |
| Sex | male |  |  | 7 |  |  | 23 |
|  | female |  |  | 6 |  |  | 15 |
| Clinical Global Impression (CGI) | | 4.54 | .66 |  | 4.61 | .79 |  |
| Global Assessment of Functioning (GAF) | | 55.31 | 4.85 |  | 55.79 | 8.39 |  |

Dimensional scales of clinical severity and psychosocial functioning in the Frankfurt sample.
